# Determinants of Total and Inhaled Allergen‐Specific Immunoglobulin E in the Middle‐Aged and Elderly Population

**DOI:** 10.1111/cea.70383

**Published:** 2026-06-27

**Authors:** M. Al Fatly, S. Leonard, P.L.A. van Daele, G. Helleman, E. Torabi‐Azhandeh, L. Lahousse, S. Veenbergen, L. Chaker

**Affiliations:** ^1^ Department of Epidemiology Erasmus University Medical Center Rotterdam the Netherlands; ^2^ Division of Endocrinology, Department of Internal Medicine Erasmus University Medical Center Rotterdam the Netherlands; ^3^ Division of Allergy & Clinical Immunology, Department of Internal Medicine Erasmus University Medical Center Rotterdam the Netherlands; ^4^ Department of Immunology, Laboratory Medical Immunology Erasmus University Medical Center Rotterdam the Netherlands; ^5^ Department of Bioanalysis Ghent University Ghent Belgium

## Abstract

- Determinants of total IgE were age, sex, smoking, BMI, topical and inhaled corticosteroids and season.
- Determinants of inhaled antigen‐specific IgE were age, sex, smoking, topical and inhaled corticosteroids and season.

To the Editor,

1

Immunoglobulin E (IgE) plays a key role in hypersensitivity reactions and immune responses against parasites and venom. While total IgE (tIgE) is generic, allergen‐specific IgE (sIgE) can confirm allergen‐specific sensitization [1]. Beyond IgE's conventionally established roles, there is increasing evidence linking tIgE and sIgE to chronic non‐communicable diseases, potentially through chronic inflammatory mechanisms [1]. Nevertheless, determinants of tIgE and sIgE remain poorly understood, especially in the general middle‐aged and elderly population, where the burden of chronic diseases is increasing.

Therefore, we utilized Rotterdam Study (RS) data to investigate age, sex, smoking, alcohol consumption, body mass index (BMI), season and corticosteroid use (topical, inhaled, oral) as potential determinants of tIgE and sIgE. A detailed description of the analyses, quality assurance, analytical considerations and results are available in an online repository (data availability statement).

The RS is a population‐based cohort including middle‐aged and elderly residents of Ommoord, Rotterdam, the Netherlands [2]. All participants provided written informed consent. We measured tIgE and sIgE between 2023–12 and 2024–11 in serum samples stored in −80°C using NOVEOS, a fully automated chemiluminescent immunoassay system [3]. The tIgE detection range was 5–5000 kU/L (kilounits/L). Serum sIgE was measured with a molecular inhalant allergen mix (SX01) in Standard Arbitrary Units (SAU). We investigated the determinants of tIgE and sIgE through multivariable linear regression analyses. We log‐transformed the outcomes and evaluated nonlinearity of age using restricted cubic splines. Lastly, we used linear mixed models to investigate longitudinal changes in tIgE and sIgE, and dose–response effects of inhaled and oral corticosteroids. We quantified the associations in odds ratios with 95% confidence intervals (OR [95% CI]).

Cross‐sectional analyses included 8679 participants (mean ± SD age 64.2 ± 9.8 years; 56.9% female), of which 478 had repeated measurements (median follow‐up 5.56 years). We identified age, sex, smoking, BMI, season and topical and inhaled corticosteroid use as determinants of tIgE and/or sIgE. Figure 1 shows the cross‐sectional and longitudinal relationships of age with tIgE and sIgE. Figures and effect estimates of other determinants are available in the online repository. First, age and tIgE showed a U‐shaped relationship (*p*‐nonlinearity < 0.001). Until approximately 70 years of age, older age was associated with lower tIgE, while after 70 years, older age was associated with higher tIgE values. For sIgE, the relationship with age was L‐shaped (*p*‐nonlinearity < 0.001), where older age was associated with lower sIgE levels and the association was most pronounced in younger ages. Both tIgE and sIgE decreased over time (*p* < 0.001). Mechanistically, the tIgE patterns may be explained by age‐related T‐ and B‐cell remodelling, including reduced IL‐4 production and impaired isotype switching to IgE [4, 5]. For sIgE, long‐term exposure to antigens throughout life could be responsible for immune tolerance against those antigens and therefore lower sIgE levels in older ages [4, 5]. Compared to men, women had lower tIgE (OR [95% CI]: 0.69 [0.65–0.74]) and sIgE (0.96 [0.92–1.00]). A possible explanation is sex hormones, contributing to variations in immune responses, antibody production and isotype switching. Sex hormones may also play a role in translating IgE sensitization into clinical manifestation of allergic symptoms. This could explain the paradoxical higher prevalence of allergy in women, despite their lower tIgE compared to men [6].

**FIGURE 1:**
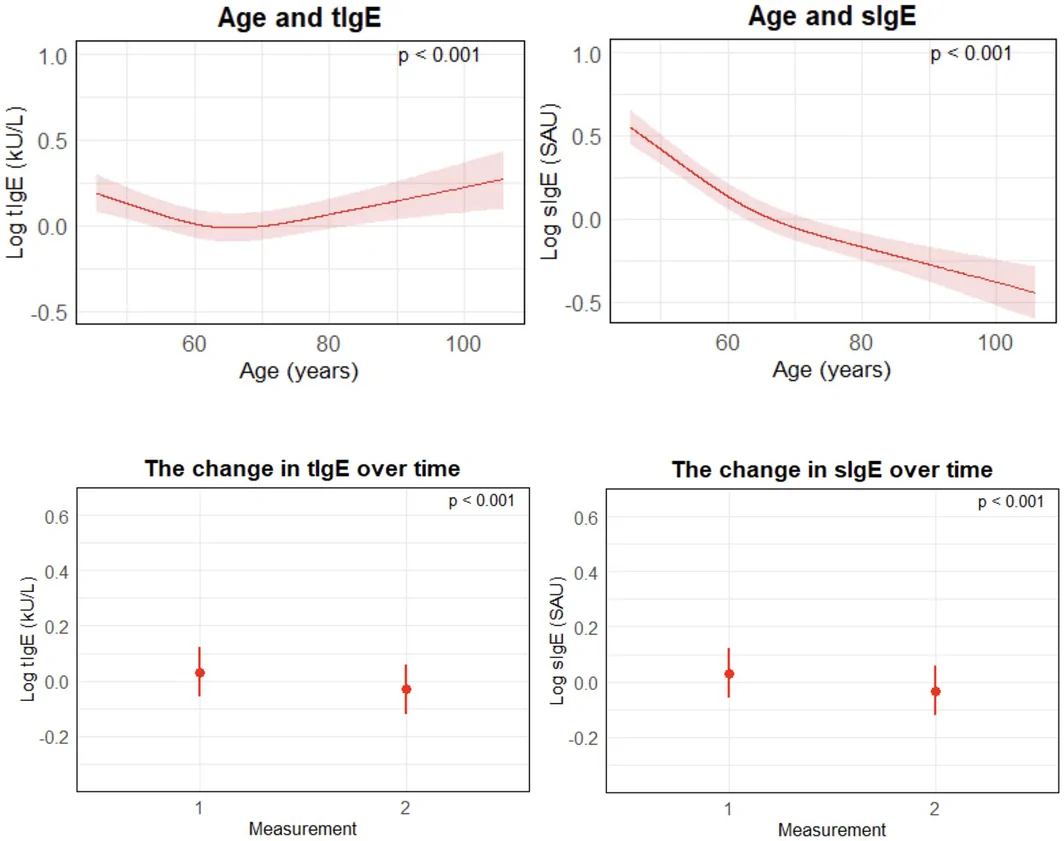
The cross‐sectional and longitudinal relationship of age with tIgE and sIgE top two panels: The cross‐sectional relationship of age with tIgE (left) and sIgE (right). Bottom two panels: The change over time in tIgE (left) and sIgE (right). kU/L, kilo units per litre; SAU, Standard Arbitrary Units; sIgE, inhaled allergen specific immunoglobulin E; TIgE, total immunoglobulin E.

Compared to never smokers, tIgE was higher in current smokers (1.34 [1.23–1.46]), whereas sIgE was lower in former (0.87 [0.83–0.91]) and current smokers (0.72 [0.68–0.76]). This pattern suggests that smoking‐related inflammation may influence total IgE production differently than allergen‐specific sensitization, and it further supports the need to interpret tIgE and sIgE as related but distinct biomarkers [7]. Conversely, alcohol consumption was not associated with tIgE or sIgE. Higher BMI was associated with higher tIgE, but not with altered sIgE. We report higher tIgE in autumn and winter compared to spring, but sIgE did not differ across seasons. However, as different allergens are increased across months, further research should account for blood sampling month and use repeated measurements to investigate intraindividual seasonal changes in tIgE/sIgE.

Lastly, topical and inhaled corticosteroids were associated with higher tIgE (topical: 1.27 [1.07–1.50], inhaled: 1.93 [1.64–2.26]) and sIgE (topical: 1.20 [1.07–1.35], inhaled: 1.64 [1.47–1.84]), while oral corticosteroid use was not. We found no dose–response effect of inhaled or oral corticosteroids. Our findings may be explained by the potential increased IgE production due to enhanced IL‐4 in corticosteroid users [8]. Our patterns suggested increases in IgE with higher inhaled corticosteroid exposure, and decreases with higher oral corticosteroid exposure. Our longitudinal findings may reflect limited power due to few corticosteroid users, confounding by indication and lack of information on timing and duration of corticosteroid use relative to blood sampling.

In conclusion, age showed a U‐shaped relationship with tIgE and L‐shaped relationship with sIgE, and both tIgE and sIgE decreased over time. Women had lower tIgE and sIgE. Current smokers had higher tIgE, whereas former and current smokers had lower sIgE. Higher BMI, and autumn and winter were associated with higher tIgE. Lastly, topical and inhaled corticosteroid use were associated with higher tIgE and sIgE, but we found no dose–response relationship. A potential limitation is prolonged storage and repeated freeze–thaw cycles of the blood samples. However, previous literature showed stability in IgE during prolonged storage when stored under ultra‐low temperatures, and our sensitivity analyses showed no batch or cohort effects [9]. A major strength is that we investigated determinants of tIgE and sIgE in a large population‐based cohort of general middle‐aged and elderly adults. The identified determinants are important to consider in clinical practice, as IgE is often interpreted using fixed thresholds without considering patient characteristics. From a research perspective, these determinants should be considered when researching IgE's role in chronic diseases and aging populations.

## Author Contributions

All persons who meet authorship criteria are listed as authors, and all authors certify that they have participated sufficiently in the work to take public responsibility for the content. Conception of the work: M. Al Fatly, S. Leonard, P.L.A. van Daele and L. Chaker. Data acquisition: G. Helleman, E. Torabi‐Azhandeh, L. Lahousse, S. Veenbergen. Statistical analysis: M. Al Fatly, S. Leonard and L. Chaker. Interpretation of the data: All authors. Writing, revision and final approval of the manuscript: All authors. Supervision and corresponding author: L. Chaker.

## Funding

The authors have nothing to report.

## Disclosure

The authors have nothing to report.

## Conflicts of Interest

The authors declare no conflicts of interest.

## Data Availability

A detailed description of the analyses, quality assurance, analytical considerations and results are available in an online repository via this link: https://doi.org/10.64898/2026.05.12.26352742.
